# Schizophrenia: Converging Neurobiological Mechanisms and Emerging Therapeutic Strategies

**DOI:** 10.3390/biom16091283

**Published:** 2026-09-04

**Authors:** Chun-Mei Gong, Kun-Ze Liu, Peng Wang, Mu-Yan Wen, Jie Wang, Zhen-Ying Li, Wei-Jingyi Lu, Zi-Liang Wang, Li-Fang Lu, Ren-Jun Feng

**Affiliations:** 1 Key Laboratory of Tropical Translational Medicine of Ministry of Education, School of Basic Medical Sciences, Hainan Academy of Medical Sciences, Hainan Medical University, Haikou 571199, China; gongchunmei@muhn.edu.cn (C.-M.G.); kunzeliu@muhn.edu.cn (K.-Z.L.); pengwang@muhn.edu.cn (P.W.); muyanwen@muhn.edu.cn (M.-Y.W.); jiewang@muhn.edu.cn (J.W.); zhenyingli@muhn.edu.cn (Z.-Y.L.); luweijingyi@muhn.edu.cn (W.-J.L.); ziliangwang@muhn.edu.cn (Z.-L.W.); 2 Key Laboratory of Brain Science Research Transformation in Tropical Environment of Hainan Province, School of Basic Medical Sciences, Hainan Academy of Medical Sciences, Hainan Medical University, Haikou 571199, China; 3 Department of Human Anatomy, School of Basic Medical Sciences, Hainan Medical University, Haikou 571199, China; 4 Department of Medical Physiology, School of Basic Medical Sciences, Hainan Medical University, Haikou 571199, China; 5 Department of Pharmacology, School of Basic Medical Sciences, Hainan Medical University, Haikou 571199, China

**Keywords:** schizophrenia, converging neurobiology, dopamine, glutamate, kynurenine pathway, neuroinflammation, gut–brain axis, precision therapeutics

## Abstract

Schizophrenia is a highly heterogeneous neuropsychiatric disorder characterized by positive symptoms, negative symptoms, and cognitive impairment, with substantial long-term functional consequences. Although dopaminergic dysfunction remains central to current disease models and treatment, dopamine-centered frameworks alone cannot fully explain cognitive deficits, treatment resistance, or marked variability in therapeutic response. Emerging evidence supports a broader view in which genetic susceptibility and environmental exposures converge on multiple interacting biological systems, including dopaminergic and glutamatergic neurotransmission, neuroimmune and glial dysfunction, kynurenine pathway metabolism, large-scale brain network dysconnectivity, and gut–brain communication. In this review, we integrate these mechanisms within a systems-level framework and discuss how their interactions may contribute to symptom heterogeneity and disease progression. We further examine the limitations of conventional dopamine D2-based antipsychotics, emerging non-dopaminergic pharmacological strategies, and adjunctive interventions targeting cognition and functional recovery. Finally, we highlight major translational barriers, including treatment resistance, medication non-adherence, adverse-effect burden, and the lack of clinically actionable biomarkers. An integrated understanding of converging neurobiological mechanisms may provide a stronger foundation for mechanism-informed patient stratification and precision treatment in schizophrenia.

## 1. Introduction

Schizophrenia is a chronic and highly heterogeneous neuropsychiatric disorder characterized by positive symptoms, negative symptoms, and cognitive impairment, all of which contribute substantially to long-term disability and poor functional outcomes [1,2]. Beyond its psychiatric and functional burden, schizophrenia is also associated with substantial premature mortality and a markedly reduced life expectancy, with excess mortality evident even among younger and middle-aged adults. This mortality gap reflects contributions from both unnatural causes, particularly suicide, and physical illnesses, including cardiovascular and metabolic diseases [3,4]. Despite decades of intensive research, the biological basis of schizophrenia remains incompletely understood, and no single pathogenic model adequately captures its remarkable clinical and molecular heterogeneity. Although dysregulated dopaminergic neurotransmission has long served as the dominant conceptual framework for schizophrenia and underpins the therapeutic efficacy of most currently available antipsychotic drugs, dopamine-centered models alone cannot satisfactorily explain persistent negative symptoms, cognitive dysfunction, treatment resistance, or the pronounced interindividual variability in clinical outcomes [5,6]. Accumulating evidence instead suggests that schizophrenia should be viewed as a disorder arising from the convergence of multiple interacting neurobiological systems rather than from dysfunction within a single neurotransmitter pathway [7,8]. Genetic susceptibility and environmental exposures jointly influence a broad spectrum of biological processes, including dopaminergic and glutamatergic neurotransmission, kynurenine pathway metabolism, neuroimmune activation, large-scale brain network organization, and gut–brain communication [9]. Importantly, these mechanisms are increasingly recognized as dynamically interconnected rather than biologically independent, collectively shaping disease onset, symptom heterogeneity, progression, and treatment responsiveness. However, despite substantial advances in each of these research areas, current knowledge remains largely fragmented. Previous reviews have often focused on individual mechanisms or specific biological domains, whereas comparatively less attention has been paid to how these systems interact across the molecular, cellular, circuit, and systemic levels to shape the complex phenotype of schizophrenia. Bridging this conceptual gap is essential for understanding why patients exhibit profound heterogeneity in symptom profiles and therapeutic responses, and for facilitating the transition from mechanism discovery to precision psychiatry. In this narrative review, we examine the etiological architecture and converging neurobiological mechanisms underlying schizophrenia across the molecular, cellular, circuit, and systemic levels. Rather than considering neurotransmitter dysfunction, immune dysregulation, metabolic alterations, brain network abnormalities, and gut–brain interactions as independent pathological events, we focus on their reciprocal relationships and their contribution to clinical heterogeneity. We further connect these mechanistic findings with emerging pharmacological and adjunctive non-pharmacological interventions, as well as major translational challenges including treatment resistance, long-term treatment limitations, and the lack of clinically actionable biomarkers. This framework aims to link advances in disease biology with the development of mechanism-informed, and ultimately, more individualized treatment strategies.

## 2. Etiological Architecture of Schizophrenia

To understand the marked clinical and biological heterogeneity of schizophrenia, it is necessary to consider its complex etiological architecture. Classical neurodevelopmental models emphasized the contribution of genetic vulnerability and early disturbances in brain development to later disease expression. This framework has subsequently evolved toward a broader developmental risk factor model in which vulnerability accumulates across multiple developmental stages through the combined influence of genetic susceptibility and environmental exposures, including obstetric complications, childhood adversity, urbanicity, migration, psychosocial stress, and cannabis use [10]. Rather than acting independently, these factors may interact across sensitive developmental windows to alter synaptic maturation, neural circuit formation, immune regulation, and neurotransmitter function, ultimately increasing susceptibility to psychosis. Schizophrenia is therefore better conceptualized as the outcome of dynamic and cumulative developmental risk rather than a disorder attributable to a single early neurodevelopmental insult. Within this framework, genetic architecture and environmental exposures represent interconnected components of disease susceptibility and provide the etiological foundation for the downstream neurobiological mechanisms discussed in Section 3.

### 2.1. Genetic Susceptibility and Molecular Risk Architecture

Genetic susceptibility constitutes the principal biological foundation of schizophrenia. Family, twin, and population-based studies consistently estimate the heritability of schizophrenia to range from approximately 60% to 80%, firmly establishing the disorder as one of the most heritable psychiatric diseases [11,12]. However, schizophrenia is not caused by mutations in a single gene. Instead, disease risk arises from a complex genetic architecture comprising numerous common variants with individually modest effects together with rare, high-impact variants that substantially increase susceptibility in a subset of patients. This polygenic architecture provides a molecular explanation for the pronounced biological and clinical heterogeneity observed across individuals with schizophrenia. Recent large-scale sequencing studies have further refined this model. A whole-exome meta-analysis involving 24,248 patients with schizophrenia and 97,322 controls identified ultra-rare coding variants in ten genes associated with markedly increased disease risk, with odds ratios ranging from 3 to 50 (*p* < 2.14 × 10^−6^) [13]. Importantly, several of these genes, including GRIN2A and GRIA3, encode components of glutamatergic signaling pathways, providing direct genetic support for the involvement of glutamatergic dysfunction in schizophrenia pathogenesis [13]. Transcriptomic and single-cell analyses have added a further layer of biological insight by demonstrating that schizophrenia-associated molecular alterations converge on higher-order functional pathways rather than isolated disease genes. Transcriptome-wide association studies have identified dysregulated gene networks within the dorsolateral prefrontal cortex, whereas recent single-cell and multi-cohort analyses have revealed pronounced cell type-specific transcriptional alterations involving both neuronal and glial populations [14,15]. Collectively, these findings indicate that schizophrenia-associated genetic variation converges on a limited number of interconnected biological systems despite remarkable heterogeneity at the individual gene level. This systems-level convergence provides a mechanistic bridge between inherited genetic risk and the downstream disturbances in neurotransmission, immune regulation, and neural circuit function discussed in subsequent sections.

### 2.2. Environmental Exposures and Gene–Environment Interplay

Although genetic predisposition establishes a vulnerable biological background, inherited susceptibility alone is insufficient to determine disease onset. Environmental exposures occurring across the prenatal, perinatal, and adolescent developmental windows substantially influence disease risk by interacting with genetically susceptible molecular pathways. These interactions affect neurodevelopment, immune regulation, neurotransmitter homeostasis, and ultimately the clinical manifestation of schizophrenia [16,17].

Among the early-life environmental factors, obstetric complications have consistently emerged as one of the strongest non-genetic risk factors for schizophrenia [18,19]. During adolescence, cannabis exposure represents another well-established environmental risk factor [20,21,22]. Importantly, the association between cannabis exposure and psychosis is modified by host genetic background. Functional polymorphisms in AKT1 have been linked to increased susceptibility to cannabis-induced psychotic symptoms, providing a representative example of biologically meaningful gene–environment interaction [23]. Collectively, these observations support a dynamic gene–environment model in which inherited susceptibility establishes a vulnerable biological background, whereas environmental exposures determine the extent, timing, and trajectory of disease expression through their effects on neurodevelopment, immune regulation, and synaptic function. This conceptual framework provides the etiological basis for the converging neurobiological mechanisms discussed in the following sections.

## 3. Converging Neurobiological Mechanisms in Schizophrenia

**Figure 1:**
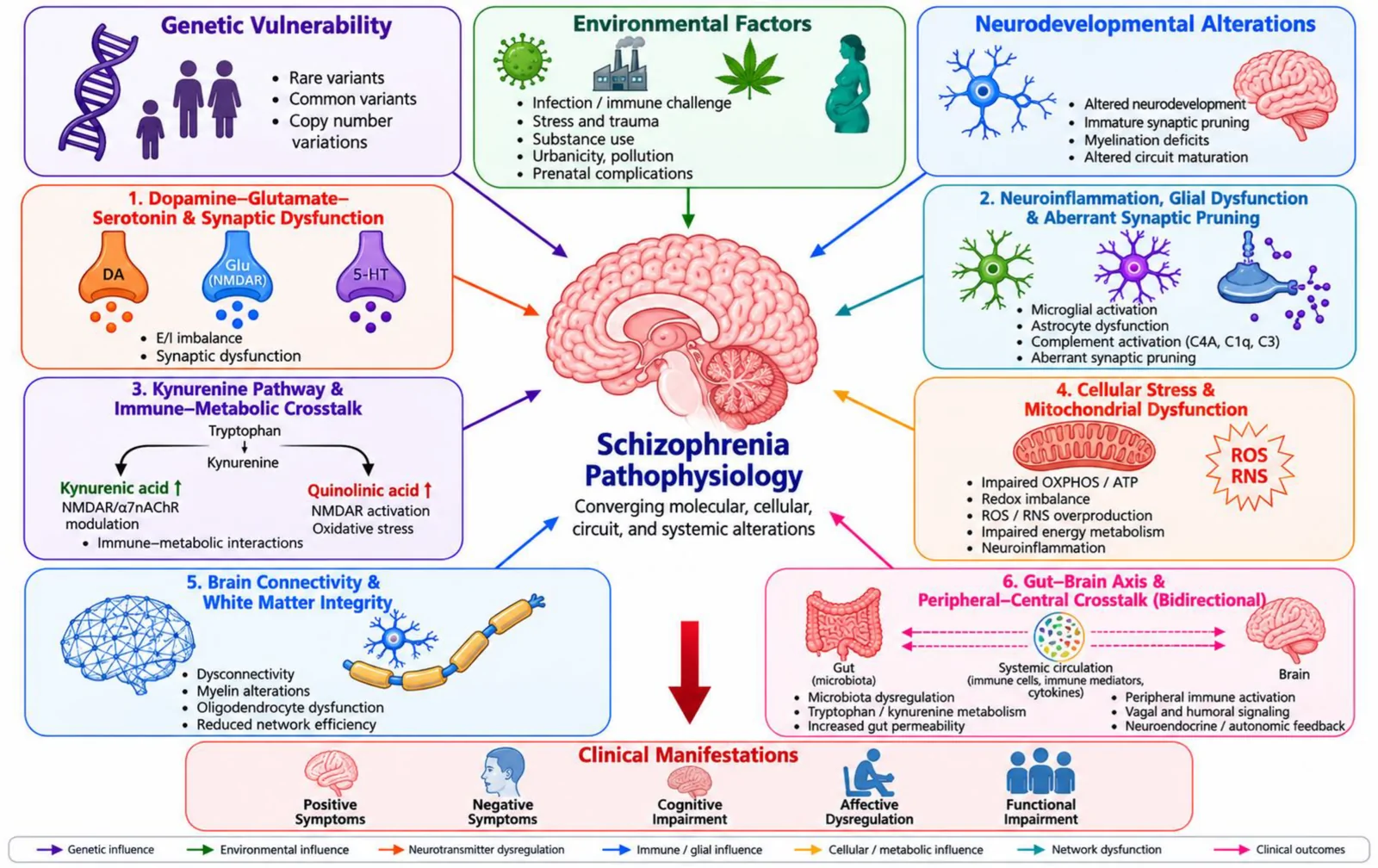
Integrated framework of converging neurobiological mechanisms in schizophrenia. Genetic susceptibility, environmental exposures, and neurodevelopmental alterations are linked to a range of biological changes, including disturbances in neurotransmission and synaptic function, neuroinflammation and glial dysfunction, aberrant synaptic pruning, kynurenine pathway alterations, cellular stress and mitochondrial dysfunction, altered brain connectivity and white matter integrity, and gut–brain signaling. These processes are likely to interact across the molecular, cellular, circuit, and systemic levels and may contribute to the clinical heterogeneity of schizophrenia, including positive and negative symptoms, cognitive impairment, affective dysregulation, and functional impairment. Abbreviations: DA, dopamine; Glu, glutamate; 5-HT, 5-hydroxytryptamine (serotonin); NMDAR, N-methyl-D-aspartate receptor; α7nAChR, α7 nicotinic acetylcholine receptor; OXPHOS, oxidative phosphorylation; ATP, adenosine triphosphate; ROS, reactive oxygen species; RNS, reactive nitrogen species; C1q, complement component 1q; C3, complement component 3; C4A, complement component 4A.

Etiological risk alone does not fully explain how schizophrenia symptoms emerge at the level of brain function. A more mechanistic account requires examining the converging neurobiological pathways through which genetic and environmental risk may be translated into neurotransmitter imbalance, immune-inflammatory dysregulation, circuit disruption, and ultimately the diverse symptom domains observed in schizophrenia. Rather than representing isolated pathogenic events, these mechanisms are increasingly understood as interacting components of a broader biological network that contributes to symptom heterogeneity, treatment response, and illness progression. The major neurobiological pathways discussed in this section are summarized in Figure 1.

### 3.1. Dopamine–Glutamate–Serotonin Interactions and Synaptic Dysfunction

The dopamine hypothesis has long occupied a central position in schizophrenia research, with excessive presynaptic dopamine synthesis and release, particularly within the striatum, being closely associated with psychotic symptom expression [24,25]. This model is strongly supported by molecular imaging studies. Meta-analyses of positron emission tomography have consistently demonstrated increased presynaptic dopamine synthesis and release in the striatum of patients with schizophrenia compared with healthy individuals [26,27]. Notably, elevated presynaptic dopamine function has also been detected during the prodromal stage, with dopamine synthesis capacity increasing further during the transition to first-episode psychosis [28]. Collectively, these findings indicate that striatal dopaminergic dysregulation represents a core neurochemical feature associated with psychosis onset. The clinical success of dopamine D2 receptor antagonists further supports the central role of dopamine signaling in schizophrenia, as therapeutic efficacy against positive symptoms closely correlates with striatal D2 receptor occupancy [24]. Nevertheless, dopamine-centered models provide only a partial explanation for disease pathophysiology. Although D2 receptor blockade effectively alleviates hallucinations and delusions in many patients, it has limited efficacy against persistent negative symptoms, cognitive impairment, and treatment-resistant schizophrenia [29,30,31].

Among these mechanisms, glutamatergic dysfunction, particularly altered N-methyl-D-aspartate receptor (NMDAR) signaling, represents one of the most extensively investigated complementary mechanisms. NMDARs expressed on cortical γ-aminobutyric acid (GABA) interneurons contribute to the maintenance of excitatory–inhibitory balance, and impaired NMDAR signaling may reduce inhibitory control over pyramidal neurons, resulting in cortical disinhibition and downstream alterations in dopaminergic activity [32,33,34]. This framework is supported by the ability of NMDAR antagonists such as ketamine and phencyclidine to reproduce positive, negative, and cognitive symptom dimensions in humans [35]. Postmortem studies have also reported alterations in NMDAR-related signaling and receptor subunit expression in cortical and hippocampal regions [36]. Furthermore, genetic studies have implicated schizophrenia risk genes involved in glutamatergic synaptic organization and NMDAR signaling [36,37]. Rather than representing competing hypotheses, dopamine and glutamate abnormalities are increasingly viewed as complementary and functionally interconnected components of a shared neurobiological network [6,38]. Altered NMDAR signaling may contribute to dopaminergic dysregulation through changes in cortical inhibitory and corticostriatal circuits, whereas abnormal dopaminergic signaling may, in turn, influence glutamatergic neurotransmission and cortical information processing. This reciprocal interaction provides a broader framework for understanding the coexistence of psychotic symptoms, cognitive impairment, and treatment heterogeneity.

Beyond receptor-level abnormalities, accumulating evidence indicates that glutamatergic dysfunction in schizophrenia is accompanied by structural alterations at excitatory synapses. Postmortem studies have consistently demonstrated reductions in dendritic spine density in pyramidal neurons of the dorsolateral prefrontal cortex (DLPFC), particularly within layer III, which contains recurrent excitatory microcircuits critical for working memory and higher-order cognitive processing [39]. Because dendritic spines constitute major postsynaptic sites of cortical excitatory input, their loss may provide a structural substrate linking impaired glutamatergic transmission to cognitive dysfunction and cortical network instability [40]. More broadly, recent evidence has led to an updated synaptic hypothesis of schizophrenia, in which genetic and environmental risk factors converge on abnormalities in synapse formation, maintenance, plasticity, and elimination [41].

Serotonergic signaling provides an additional modulatory layer within this interconnected neurotransmitter network. In particular, 5-HT2A and 5-HT1A receptors can modulate cortical glutamatergic and dopaminergic neurotransmission and contribute to the pharmacological actions of several atypical antipsychotics [42,43]. Importantly, these receptor subtypes have distinct pharmacological roles: 5-HT2A receptor antagonism is a characteristic serotonergic action of many atypical antipsychotics, whereas 5-HT1A receptor agonism or partial agonism is present in some agents and has also been investigated as a complementary mechanism in schizophrenia treatment [44]. Rather than constituting an independent pathogenic pathway, serotonergic dysfunction may therefore be better viewed as part of a broader regulatory network interacting with dopamine and glutamate systems. Interactions between serotonergic and glutamatergic signaling, including functional crosstalk involving 5-HT2A and metabotropic glutamate receptors, further illustrate the interconnected nature of these neurotransmitter systems [45]. This perspective is also therapeutically relevant because several emerging or recently investigated pharmacological strategies engage serotonergic signaling alongside non-D2 targets, as discussed in Section 4.

Importantly, synaptic abnormalities are unlikely to arise exclusively from neuronal dysfunction. Increasing evidence indicates that immune and glial pathways participate in synaptic remodeling, with complement signaling and microglial phagocytosis emerging as candidate mechanisms linking genetic and inflammatory risk to excessive synaptic elimination [41,46]. Patient-derived cellular studies and convergent experimental evidence further suggest that complement-dependent microglial engulfment may contribute to abnormal synaptic loss in schizophrenia [41]. This neuroimmune–synaptic interface therefore provides a mechanistic bridge between neurotransmitter dysfunction and the glial, inflammatory, and metabolic abnormalities discussed in the following sections.

### 3.2. Neuroinflammation, Glial Dysfunction, and Aberrant Synaptic Pruning

Increasing evidence indicates that immune dysregulation and glial dysfunction constitute an important interface between neurodevelopmental vulnerability and synaptic abnormalities in schizophrenia. Alterations in circulating inflammatory mediators have been repeatedly reported across different stages of the disorder, although their magnitude and direction vary according to illness stage, treatment status, and other clinical factors [47]. Importantly, inflammatory abnormalities have also been detected in young adults with first-episode psychosis, including antipsychotic-naïve individuals, indicating that immune dysregulation can already be present around the onset of illness rather than emerging exclusively during chronic disease [48,49]. However, peripheral inflammatory alterations should not be equated directly with central neuroinflammation, for which in vivo evidence remains heterogeneous. Within the central nervous system, microglia, astrocytes, and oligodendrocytes are increasingly recognized not merely as supporting cells but as active regulators of synaptic maturation, neurotransmitter homeostasis, metabolic support, myelination, and neuroimmune signaling [50]. Dysregulation of these interconnected glial functions may therefore provide a cellular mechanism through which inflammatory and developmental risk factors are translated into synaptic and circuit abnormalities.

A particularly compelling link between immune dysregulation and synaptic pathology involves the classical complement system. Genetic studies have identified structural variation at the complement component 4 (C4) locus as a major schizophrenia-associated signal, with increased C4A expression associated with greater disease risk. During normal neurodevelopment, complement proteins participate in activity-dependent synaptic refinement: C1q and downstream C3 can tag synaptic elements, which can subsequently be recognized by complement receptor 3 (CR3)-expressing microglia and targeted for elimination. Dysregulation of this machinery has therefore been proposed to contribute to aberrant synaptic remodeling in schizophrenia [41,51,52]. Supporting this model, patient-derived cellular systems have demonstrated increased microglia-mediated synapse elimination, with schizophrenia-associated C4 variants linked to increased neuronal complement deposition and synaptic uptake [53]. Recent experimental work further supports a functional C4–CR3 connection in regulating cortical synapse density while also indicating that the effects of C4 dysregulation on microglia–synapse interactions may be more complex than a simple increase in phagocytic pruning [54]. Thus, complement-mediated synaptic remodeling represents a plausible mechanistic bridge between genetic risk, innate immune signaling, and the synaptic deficits observed in schizophrenia, although its precise contribution in the human disorder remains to be fully defined.

Beyond complement-dependent synaptic remodeling, microglial dysfunction may contribute to schizophrenia through broader inflammatory and plasticity-related mechanisms. Activated or dysregulated microglial states can alter the production of inflammatory mediators, influence glutamatergic synapses, and modify neuronal plasticity. Experimental and patient-derived evidence suggests that abnormal microglia–neuron interactions may preferentially affect cortical glutamatergic synapses, providing a potential connection between the inflammatory environment and the glutamatergic and dendritic spine abnormalities described above [41,46]. Importantly, however, microglial abnormalities in schizophrenia are heterogeneous across experimental models, brain regions, illness stages, and patient populations. Neuroinflammation should therefore not be interpreted as a uniform state present in all patients, but rather as a potentially important biological dimension in a subset of schizophrenia phenotypes [47]. Astrocytes represent a second major component of this neuroimmune–synaptic interface. In addition to regulating extracellular glutamate through uptake and recycling, astrocytes support synaptogenesis, metabolic homeostasis, blood–brain barrier function, and neuronal energy metabolism. They can also influence complement-dependent synaptic refinement by regulating complement-related signaling and interacting with microglial pruning pathways. Evidence from postmortem, genetic, transcriptomic, and experimental studies suggests abnormalities in astrocyte maturation and function in schizophrenia, with potential consequences for glutamatergic and dopaminergic neurotransmission, mitochondrial function, and synaptic development [55,56]. Astrocyte dysfunction may therefore connect neurotransmitter abnormalities with immune activation and metabolic stress rather than representing an isolated glial pathology. Oligodendrocyte abnormalities add another dimension to glial dysfunction in schizophrenia. Beyond generating myelin, oligodendrocytes provide metabolic and trophic support to axons and are essential for the temporal coordination of information transfer across distributed neural networks. Transcriptomic, postmortem, and neuroimaging evidence has identified altered oligodendrocyte-related gene expression, oligodendrocyte morphology, and myelination in schizophrenia [57]. Such abnormalities may impair axonal conduction and long-range network synchronization, providing a cellular substrate for the white matter and connectivity disturbances discussed in Section 3.5. Importantly, inflammatory and oxidative environments may also interfere with oligodendrocyte maturation and myelin maintenance, suggesting that immune dysregulation, glial dysfunction, and network dysconnectivity may be mechanistically interconnected rather than independent pathological processes. Glial and immune abnormalities may also intersect with redox dysregulation. Oxidative and nitrosative stress can arise when the generation of reactive oxygen and nitrogen species exceeds antioxidant capacity, potentially affecting mitochondrial function, membrane lipids, synaptic proteins, and cellular signaling. Glutathione, a major intracellular antioxidant, has therefore received considerable attention in schizophrenia, although in vivo magnetic resonance spectroscopy studies show substantial heterogeneity across clinical stages and brain regions [58]. Redox imbalance may be particularly relevant to fast-spiking interneurons, oligodendrocytes, and metabolically demanding synaptic processes, while inflammatory signaling can further amplify oxidative stress. These reciprocal interactions suggest that neuroinflammation and redox dysfunction may form a feed-forward process contributing to impaired synaptic plasticity and circuit stability rather than representing independent disease mechanisms [59]. Collectively, these findings position glial cells at the intersection of immune signaling, synaptic remodeling, neurotransmitter homeostasis, redox regulation, and network connectivity. Within this framework, immune activation may also reshape tryptophan metabolism through the kynurenine pathway, generating neuroactive metabolites that directly influence glutamatergic signaling and inflammatory responses. The kynurenine pathway therefore represents an additional immune–metabolic mechanism through which peripheral and central inflammatory processes may be translated into neuronal dysfunction.

### 3.3. Kynurenine Pathway and Immune–Metabolic Crosstalk

The kynurenine pathway provides an important metabolic interface between immune activation, glial function, and neurotransmitter dysregulation in schizophrenia. As the principal route of tryptophan degradation, this pathway generates several neuroactive metabolites, among which kynurenic acid (KYNA) and quinolinic acid (QUIN) have received particular attention because of their effects on glutamatergic signaling, inflammatory responses, and neuronal function [60,61,62]. Within the brain, kynurenine metabolism is compartmentalized across different cell types: astrocytes are considered a major source of KYNA, whereas activated microglia and infiltrating macrophages preferentially generate metabolites along the 3-hydroxykynurenine (3-HK)/QUIN branch. Consequently, inflammatory changes in glial state may shift kynurenine metabolism and thereby provide a direct metabolic link between the neuroimmune abnormalities described above and altered neurotransmission. KYNA is particularly relevant to schizophrenia because it can modulate both NMDAR and α7 nicotinic acetylcholine receptor (α7nAChR) signaling. Increased KYNA concentrations have been reported in cerebrospinal fluid and postmortem brain tissue from patients with schizophrenia, although findings vary across cohorts, disease stages, and biological compartments [63,64,65]. Several mechanisms may contribute to this elevation. Increased availability of kynurenine, including that arising from inflammation-associated activation of tryptophan metabolism, may provide more substrate for cerebral KYNA synthesis, while reduced kynurenine 3-monooxygenase (KMO) activity may divert kynurenine away from the 3-HK branch toward KYNA production in astrocytes [66,67]. Thus, elevated KYNA is more likely to reflect altered pathway flux and the balance of kynurenine-metabolizing enzymes than simple overproduction through a single mechanism. Excessive KYNA-mediated inhibition of glutamatergic and cholinergic signaling may contribute to impaired working memory, attention, and executive function, thereby providing a potential metabolic mechanism linking immune–glial dysfunction to the cognitive abnormalities discussed in Section 3.1 [68]. Importantly, KYNA should not be regarded as uniformly neuroprotective or detrimental. Its biological effects are concentration- and context-dependent, and physiological KYNA signaling may protect against excessive excitatory activity, whereas sustained elevations may interfere with receptor signaling required for normal cognition and synaptic plasticity.

In contrast, QUIN is generated predominantly through the microglial/macrophage branch of kynurenine metabolism and can activate NMDARs while promoting oxidative and inflammatory stress. Excessive QUIN accumulation may therefore contribute to excitotoxicity and neuronal dysfunction [69]. Nevertheless, the functional significance of individual kynurenine metabolites cannot be inferred simply from a binary “neuroprotective versus neurotoxic” classification. Rather, the balance among KYNA, QUIN, 3-HK, and other pathway intermediates is influenced by immune activation, cellular source, metabolic enzyme activity, and disease context. Alterations in this balance may consequently produce different effects on glutamatergic transmission, redox homeostasis, and neuronal function [70,71]. Taken together, kynurenine pathway dysregulation provides a plausible biochemical bridge linking immune activation and glial dysfunction to neurotransmitter and cognitive abnormalities in schizophrenia. Rather than constituting an isolated metabolic pathway, it may form part of a broader immune–metabolic network in which inflammatory signaling reshapes tryptophan metabolism, while neuroactive kynurenine metabolites feedback on glutamatergic transmission, oxidative balance, and neural plasticity. This framework further illustrates how molecular and cellular disturbances may propagate beyond local biochemical changes to influence distributed brain circuits and connectivity.

### 3.4. Cellular Stress and Mitochondrial Dysfunction

Mitochondrial and cellular stress pathways represent another potential point of convergence among the neurodevelopmental, inflammatory, metabolic, and synaptic abnormalities implicated in schizophrenia. Neurons have exceptionally high energetic demands, particularly for maintaining membrane potentials, neurotransmitter cycling, axonal transport, and synaptic plasticity, making neuronal function highly dependent on mitochondrial bioenergetic capacity. Accumulating evidence from postmortem brain tissue, neuroimaging, peripheral samples, and patient-derived cellular models suggests alterations in mitochondrial function and energy metabolism in schizophrenia, although the magnitude and direction of these abnormalities vary across tissues, disease stages, and treatment conditions [72,73]. Rather than representing an isolated metabolic defect, mitochondrial dysfunction may therefore constitute a cellular interface through which multiple upstream disturbances influence neuronal and synaptic function.

Impaired oxidative phosphorylation (OXPHOS) is particularly relevant to this framework. Alterations in mitochondrial respiratory-chain activity, including abnormalities involving complex I and other OXPHOS components, have been reported in schizophrenia and may compromise ATP production required for synaptic transmission and neuronal homeostasis [73]. Bioenergetic impairment may be especially consequential for metabolically demanding processes such as glutamatergic neurotransmission, interneuron function, synaptic remodeling, and myelin maintenance. In this context, mitochondrial abnormalities provide a plausible mechanistic link between cellular energy failure and the synaptic and circuit disturbances described in the preceding sections. Importantly, however, mitochondrial findings in schizophrenia remain heterogeneous and can be influenced by medication exposure, smoking, metabolic comorbidity, tissue source, and illness duration; mitochondrial dysfunction should therefore be considered a convergent biological dimension rather than a universal primary defect.

Mitochondrial dysfunction is also closely intertwined with redox and inflammatory stress. Impaired respiratory-chain function can alter reactive oxygen species generation, whereas insufficient antioxidant capacity may increase the vulnerability of cellular membranes, proteins, and synaptic structures to oxidative damage. Conversely, inflammatory signaling can disturb mitochondrial metabolism, creating the potential for reciprocal interactions among mitochondrial dysfunction, oxidative stress, and neuroinflammation. Recent clinical evidence further supports this intersection: mitochondrial alterations have been associated with cognitive impairment in schizophrenia, with inflammatory status potentially modifying this relationship [74]. Emerging work also suggests that redox disturbance may extend beyond conventional oxidative stress to include altered reducing-equivalent balance and NAD^+^/NADH homeostasis, linking mitochondrial bioenergetic dysfunction to broader cellular stress responses [75].

Beyond mitochondrial bioenergetics, broader metabolic reprogramming may also be relevant to schizophrenia and its treatment. Alterations in glucose, lipid, amino acid, and nucleotide metabolism may interact with redox regulation, immune signaling, and neurotransmitter homeostasis, suggesting that metabolic networks could represent modifiable components of the disease-associated biological state. Recent evidence from electroconvulsive therapy (ECT) further suggests that therapeutic response may be accompanied by changes in metabolic pathways linked to neural, immune, and neuroendocrine regulation, although whether such metabolic changes directly mediate clinical improvement remains unclear [76].

Taken together, mitochondrial dysfunction and cellular stress may amplify rather than independently initiate schizophrenia-related pathology. Energy failure, redox imbalance, and inflammatory signaling can potentially impair synaptic plasticity and neurotransmission, while persistent synaptic and inflammatory disturbances may in turn increase cellular energetic demands and mitochondrial stress. This reciprocal relationship provides another mechanism through which diverse upstream risk factors may converge on neuronal dysfunction and cognitive impairment. Nevertheless, mitochondrial and metabolic abnormalities are unlikely to define all patients with schizophrenia, and their value as biomarkers or therapeutic targets will require validation in biologically stratified populations.

### 3.5. Brain Connectivity and White Matter Integrity

Schizophrenia is increasingly conceptualized as a disorder of large-scale brain network dysconnectivity rather than dysfunction confined to a single neurotransmitter system or discrete anatomical region [77,78,79]. From this perspective, abnormalities in synaptic organization, excitatory–inhibitory balance, and myelination may converge to disrupt coordinated communication across the cortical and subcortical circuits. In particular, NMDAR hypofunction and impaired GABAergic interneuron activity may disturb neuronal synchronization and cortical information processing, providing a potential molecular-to-circuit link between the neurotransmitter abnormalities described above and large-scale network dysfunction [80]. Structural and diffusion neuroimaging studies provide substantial evidence for abnormalities in white matter organization in schizophrenia. Large-scale diffusion magnetic resonance imaging analyses have identified widespread reductions in fractional anisotropy, with prominent abnormalities involving major association and commissural pathways, supporting impaired structural connectivity across distributed brain regions [81]. These alterations are not restricted to chronic illness; abnormalities in white matter organization and developmental trajectories have also been reported in individuals at clinical high risk and during the early stages of psychosis [82]. This suggests that at least some white matter abnormalities may emerge before or around illness onset rather than solely representing a consequence of aging or chronic disease. However, white matter changes may continue to evolve over the course of schizophrenia and can be influenced by aging, illness duration, treatment exposure, and other clinical factors [81,83]. Accordingly, current evidence does not establish white matter dysfunction as a primary etiological factor, and the observed abnormalities may reflect a combination of neurodevelopmental vulnerability and secondary or progressive changes. Importantly, these imaging abnormalities may have a cellular correlate in oligodendrocyte and myelin dysfunction. Altered expression of myelination-related genes, together with postmortem evidence of oligodendrocyte abnormalities, supports the possibility that impaired myelin formation and maintenance contribute to inefficient long-range neuronal communication in schizophrenia [57]. Functional neuroimaging further indicates that schizophrenia is characterized by altered communication both within and between large-scale networks, particularly the default mode network, salience network, and central executive network [84,85]. These systems contribute to internally directed cognition, salience attribution, executive control, and the integration of external and internal information. Their disrupted coordination may therefore contribute to cognitive impairment, abnormal salience processing, and other symptom dimensions of schizophrenia. The amygdala is also an important component of these distributed circuits, particularly in networks involved in emotional processing, salience attribution, and social cognition. Structural and functional alterations of the amygdala have been reported in schizophrenia, with some studies linking these abnormalities to negative symptoms and impaired social functioning [86,87]. Rather than representing an isolated pathological feature, amygdala dysfunction may therefore contribute to schizophrenia through altered interactions with prefrontal and other limbic regions. More broadly, the negative symptoms of schizophrenia cannot be attributed simply to neuronal loss in a specific brain region. Current evidence instead points to abnormalities across prefrontal–striatal–limbic circuits, involving altered synaptic organization, neurotransmitter signaling, and network function [88,89]. Although structural changes have also been reported, widespread neuronal loss has not been established as the primary pathological basis of negative symptoms. However, dysconnectivity patterns are heterogeneous and can vary with illness stage, medication exposure, symptom profile, and analytical methodology; accordingly, no single connectivity signature currently defines schizophrenia at the individual-patient level. Collectively, structural and functional dysconnectivity may represent a systems-level manifestation of multiple upstream abnormalities rather than an independent pathogenic mechanism. Synaptic loss, altered glutamatergic–GABAergic signaling, glial and myelin dysfunction, neuroinflammatory processes, and metabolic disturbances may all converge on impaired circuit organization and communication. This systems-level perspective therefore provides an important bridge between molecular and cellular pathology and the heterogeneous cognitive and clinical manifestations of schizophrenia. At the same time, brain network function may also be influenced by signals originating outside the central nervous system, including immune and metabolic inputs from the gut–brain axis, which are considered in the following section.

### 3.6. Gut–Brain Axis Disturbance and Peripheral–Central Immune Crosstalk

The gut–brain axis has emerged as a potentially important, although still evolving, component of schizophrenia pathophysiology. Human studies have identified differences in gut microbial composition between individuals with schizophrenia and healthy controls, but the specific taxonomic patterns reported across studies remain heterogeneous [90,91]. A recent meta-analysis further indicated that schizophrenia and antipsychotic exposure may exert both distinct and overlapping effects on gut microbial composition, emphasizing the importance of medication and other clinical confounders when interpreting microbiome findings [92]. Thus, current evidence supports the presence of schizophrenia-associated microbial alterations at the group level, but does not yet define a disease-specific microbial signature. Several biological routes may connect gut microbial alterations with central nervous system function. Microbial metabolites, intestinal barrier function, immune signaling, and tryptophan metabolism can influence systemic inflammatory tone and potentially modify neuroimmune and neurotransmitter pathways. In particular, gut-derived microbial products and altered barrier integrity may promote peripheral innate immune signaling, whereas microbial metabolites such as short-chain fatty acids and tryptophan derivatives can modulate immune and metabolic homeostasis. These peripheral signals may subsequently influence blood–brain barrier function and glial activity, providing a potential route through which gut dysbiosis could interact with the neuroinflammatory mechanisms described in Section 3.2. However, direct evidence establishing a causal sequence from intestinal dysbiosis to microglial activation in patients with schizophrenia remains limited, and this relationship should therefore be interpreted cautiously. Importantly, communication along the gut–brain axis is bidirectional. Changes in central neural activity, autonomic and neuroendocrine signaling, and stress-related physiology may also influence gastrointestinal function and the intestinal microbial environment [93,94]. Accordingly, the microbial alterations observed in schizophrenia may represent not only potential contributors to disease-related processes, but also consequences or modifiers of central and systemic changes associated with the disorder.

The kynurenine pathway provides a particularly relevant metabolic interface between the gut and the neurobiological mechanisms discussed above. Gut microorganisms can influence host tryptophan availability and metabolism, potentially altering the balance among serotonin, kynurenine, and microbially derived indole metabolites [95]. Experimental evidence supports the biological plausibility of this connection: transplantation of gut microbiota from patients with schizophrenia into mice has been reported to induce schizophrenia-relevant behavioral abnormalities together with alterations in tryptophan–kynurenine metabolism and neurotransmitter signaling [96]. A separate microbiota-transfer study demonstrated alterations in the glutamate–glutamine–GABA cycle and schizophrenia-relevant behaviors in recipient mice [97]. These findings provide experimental support for microbiome–metabolite–brain interactions, although their translation to human schizophrenia remains uncertain.

Collectively, the gut–brain axis may represent a bidirectional interface linking central neural processes with peripheral immune, metabolic, and microbial signals rather than an independent pathogenic pathway in schizophrenia. Its potential interactions with inflammatory signaling, glial function, kynurenine metabolism, and excitatory–inhibitory neurotransmission further reinforce the concept that schizophrenia arises from converging biological disturbances across multiple levels of organization. Nevertheless, substantial interindividual variability and potential confounding by diet, lifestyle, medication exposure, metabolic status, and geography currently limit the use of microbiome profiles as diagnostic or predictive biomarkers. Future longitudinal and mechanistically informed studies will be required to determine whether microbiome alterations contribute causally to specific schizophrenia phenotypes and whether they can be translated into clinically meaningful therapeutic targets.

## 4. Translational Implications for Treatment

**Figure 2:**
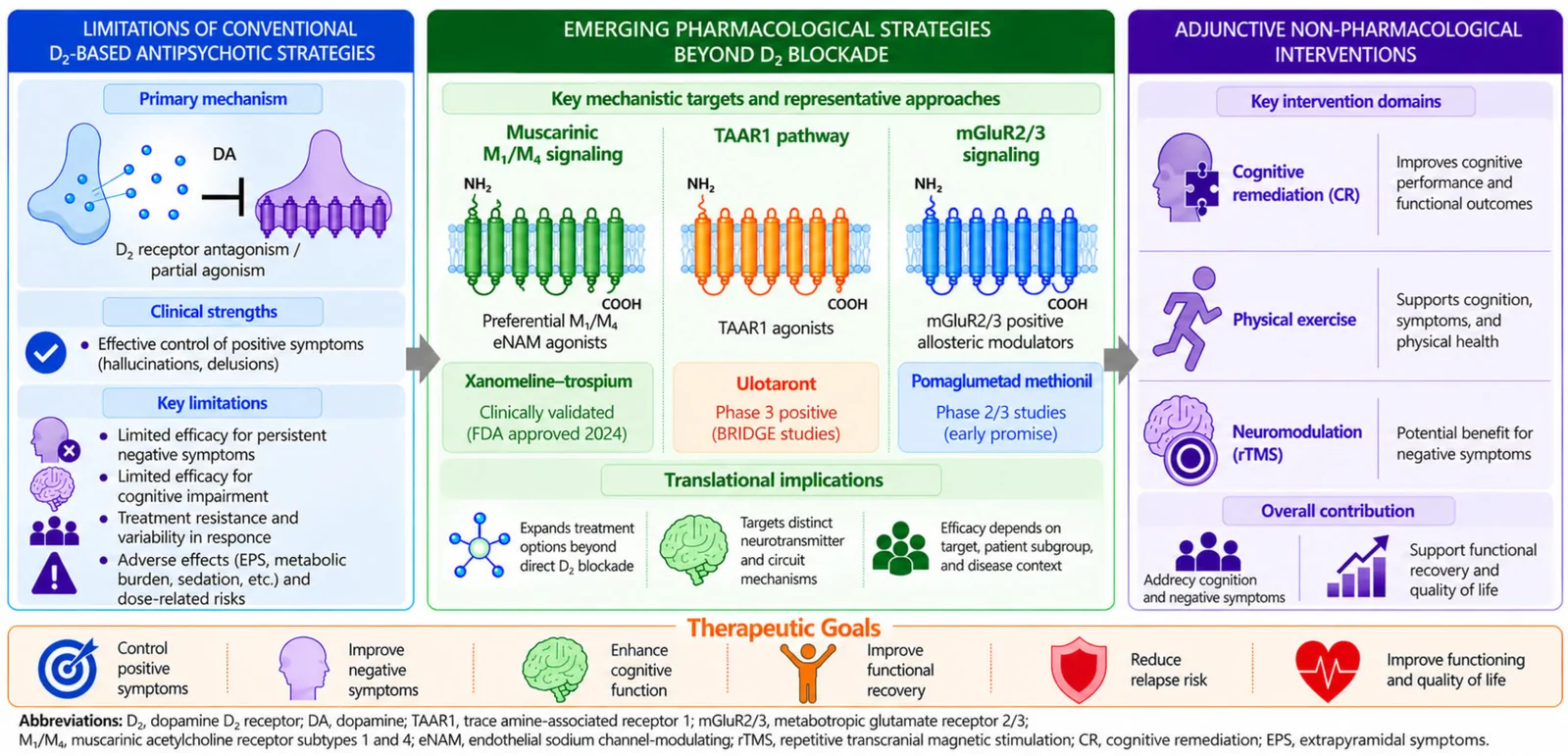
Mechanism-informed therapeutic strategies for schizophrenia. Conventional antipsychotic treatment remains largely dependent on dopamine D_2_ receptor antagonism or partial agonism and is most effective for positive symptoms, while persistent negative symptoms, cognitive impairment, treatment resistance, and adverse effects remain important limitations. Pharmacological approaches beyond direct D_2_ receptor blockade include muscarinic M_1_/M_4_ receptor modulation, TAAR1 agonism, and mGluR2/3-targeted strategies, which have shown varying levels of clinical evidence and translational success. Adjunctive non-pharmacological interventions, including cognitive remediation, physical exercise, and repetitive transcranial magnetic stimulation (rTMS), may provide additional benefits for cognition, negative symptoms, and functional recovery. Together, these approaches reflect an expanding therapeutic framework aimed at addressing a broader range of symptoms and functional outcomes in schizophrenia.

The convergent biological framework outlined above has important implications for the treatment of schizophrenia. Although dopamine D2 receptor blockade remains central to antipsychotic pharmacotherapy, the involvement of glutamatergic, cholinergic, serotonergic, immune–metabolic, and cellular stress pathways suggests that therapeutic development should extend beyond dopamine signaling alone. This broader perspective is particularly relevant to persistent negative symptoms, cognitive impairment, treatment resistance, and functional disability, which remain inadequately addressed by conventional antipsychotic treatment. Accordingly, recent therapeutic development has increasingly shifted toward mechanistically diversified pharmacological strategies, alongside adjunctive interventions targeting cognition and functional recovery. The major therapeutic strategies discussed in this section are summarized in Figure 2.

### 4.1. Limitations of Conventional D2-Based Antipsychotic Strategies

Antipsychotic medications remain the cornerstone of schizophrenia treatment and are effective in reducing acute psychotic symptoms and preventing relapse in many patients [98]. Despite substantial pharmacological diversity, the therapeutic actions of most conventional antipsychotics remain closely linked to dopamine D2 receptor antagonism or partial agonism [6]. Molecular imaging and pharmacological studies have demonstrated a relationship between striatal D2 receptor occupancy and antipsychotic efficacy, supporting the continued clinical relevance of dopaminergic mechanisms. However, this relationship also illustrates an important therapeutic constraint: increasing D2 receptor occupancy beyond the range associated with optimal antipsychotic efficacy can increase the risk of extrapyramidal adverse effects and other dopamine-related complications [99].

More importantly, D2-centered pharmacotherapy addresses only part of the clinical spectrum of schizophrenia. Conventional antipsychotics are generally more effective against positive symptoms than against persistent negative symptoms and cognitive impairment, which are major determinants of long-term disability and functional outcome [6,100]. Treatment response also varies considerably among individuals, and a substantial proportion of patients show inadequate improvement despite appropriate antipsychotic treatment [101]. These limitations are consistent with the broader neurobiological framework discussed above, in which psychosis-related dopaminergic abnormalities coexist with disturbances in glutamatergic and cholinergic signaling, synaptic plasticity, immune–metabolic regulation, and distributed neural circuits. Thus, the modulation of dopamine signaling alone is unlikely to normalize the full range of biological abnormalities underlying schizophrenia.

The clinical limitations of D2-based treatment are further compounded by adverse-effect burden. First-generation antipsychotics are generally associated with a greater liability for extrapyramidal symptoms, whereas several second-generation agents carry substantial risks of weight gain, metabolic dysfunction, sedation, and other treatment-limiting adverse effects [98]. Importantly, these profiles differ considerably among individual drugs, making a simple first-generation versus second-generation distinction insufficient to predict tolerability [98]. Dose is also relevant: a systematic review and dose–response meta-analysis involving more than 37,000 participants found that the risk of extrapyramidal adverse effects generally increased with antipsychotic dose and rose substantially at estimated D2 receptor occupancies above approximately 75–85% [99].

Newer D2 partial agonists, including aripiprazole, brexpiprazole, and cariprazine, provide greater pharmacodynamic flexibility and may offer advantages in selected symptom domains or adverse-effect profiles [98,102]. Nevertheless, they remain fundamentally embedded within dopamine-centered pharmacotherapy and do not fully overcome persistent cognitive impairment, negative symptoms, or treatment resistance. These therapeutic gaps have provided a strong rationale for developing pharmacological strategies that engage mechanisms outside direct D2 receptor blockade.

### 4.2. Emerging Pharmacological Strategies Beyond Dopamine Blockade

The limitations of conventional D2 receptor-based treatment have stimulated the development of pharmacological strategies that modulate schizophrenia-relevant circuits without directly blocking dopamine D2 receptors [103]. Among these approaches, muscarinic acetylcholine receptor modulation has achieved the clearest recent clinical translation [104,105,106]. M1 and M4 receptors are G-protein-coupled receptors with distinct but complementary functions: M1 receptors are predominantly coupled to Gq/11 signaling and are widely expressed in cortical and hippocampal regions involved in cognition and synaptic plasticity, whereas M4 receptors are primarily Gi/o-coupled and are highly expressed in the striatum, where their activation can indirectly regulate dopaminergic signaling [103,104]. This functional organization provides a mechanistic rationale for targeting M1/M4 receptors to influence both cognitive–cortical and dopaminergic–striatal circuits without direct D2 receptor antagonism.

Xanomeline is a muscarinic agonist with preferential functional activity at the M1 and M4 receptors. Its clinical development was historically limited by peripheral cholinergic adverse effects; this limitation was addressed by combining xanomeline with trospium chloride, a peripherally restricted muscarinic antagonist designed to reduce peripheral cholinergic effects while preserving central muscarinic activity. In the phase 3 EMERGENT-2 trial, xanomeline–trospium produced a significantly greater reduction in Positive and Negative Syndrome Scale (PANSS) total scores than the placebo after five weeks, with a between-group difference of 9.6 points [106]. Similar efficacy was observed in EMERGENT-3 [105], and pooled analyses of the EMERGENT trials further supported improvement across schizophrenia symptom domains [107]. These findings led to the approval of xanomeline–trospium in the United States in September 2024 for the treatment of schizophrenia in adults, representing the first approved antipsychotic approach based primarily on cholinergic rather than direct dopamine receptor targeting. Nevertheless, gastrointestinal and other cholinergic adverse events remain relevant, and longer-term comparative effectiveness and real-world tolerability require continued evaluation.

Trace amine-associated receptor 1 (TAAR1) represents another mechanistically distinct therapeutic target [108]. TAAR1 is an intracellularly located G-protein-coupled receptor expressed in monoaminergic brain regions and can regulate dopaminergic signaling through interactions with dopamine transporter function, presynaptic neuronal activity, and downstream intracellular signaling. TAAR1 activation may also influence serotonergic and glutamatergic neurotransmission, suggesting a broader modulatory role than direct D2 receptor blockade [108,109]. Ulotaront (SEP-363856) [110], an agonist with activity at the TAAR1 and 5-HT1A receptors, initially showed significant improvement in overall schizophrenia symptoms in a phase 2 trial and generated particular interest because of its limited liability for extrapyramidal and metabolic adverse effects. Preclinical evidence further suggests that TAAR1 agonism can modulate striatal and hippocampal glutamatergic transmission in a state-dependent manner [111].

However, subsequent clinical findings have tempered the initial enthusiasm surrounding TAAR1 agonism. A recent systematic review and meta-analysis incorporating randomized trials of ulotaront and ralmitaront found only small and statistically uncertain improvements in overall symptoms compared with the placebo in acute schizophrenia, with substantial placebo responses contributing to the negative phase 3 findings [112]. These results do not necessarily invalidate TAAR1 biology, but they illustrate an important translational challenge: pharmacological modulation of a biologically plausible target does not guarantee clinically meaningful efficacy across a heterogeneous diagnostic population. Differences in disease stage, symptom dimensions, underlying neurobiology, target engagement, and trial design may all influence treatment response.

Glutamatergic strategies provide an even clearer example of this translational gap. Given the substantial evidence implicating NMDAR hypofunction and altered glutamatergic signaling in schizophrenia, considerable effort has been directed toward enhancing NMDAR function or regulating glutamate release. Group II metabotropic glutamate receptor (mGluR2/3) agonists were particularly attractive because the activation of these receptors can reduce excessive glutamate release and indirectly influence downstream dopaminergic activity. Pomaglumetad methionil (LY2140023), a prodrug of an mGluR2/3 agonist, showed encouraging early findings but subsequently failed to demonstrate consistent antipsychotic efficacy in larger phase 2 and phase 3 studies [113,114]. More recent evidence continues to show no consistent overall therapeutic benefit, although subgroup and biomarker-based approaches remain of interest.

The experience with mGluR2/3 modulation is particularly informative for mechanism-based drug development in schizophrenia. Failure may reflect not only insufficient target efficacy but also biological heterogeneity within schizophrenia, differences between mGluR2 and mGluR3 signaling, disease-stage dependence, previous antipsychotic exposure, inadequate target engagement, and the absence of biomarkers capable of identifying responsive subgroups. Indeed, post hoc analyses of pomaglumetad trials suggested possible differential responses according to illness duration and genetic background, although these findings have not established a clinically validated responder population [114]. Recent reassessments of mGluR2 drug development similarly emphasize patient stratification and response-predictive biomarkers as prerequisites for revisiting this pathway.

Beyond these relatively advanced approaches, the convergent mechanisms described in Section 3 suggest additional therapeutic opportunities involving immune–inflammatory signaling, kynurenine metabolism, redox and mitochondrial homeostasis, and microbiota-related pathways. Among these, antioxidant and redox-modulating interventions, particularly N-acetylcysteine (NAC), have been investigated as adjunctive strategies based on evidence of oxidative and mitochondrial abnormalities in schizophrenia. Meta-analytic findings, however, have been inconsistent, with some analyses reporting improvements in psychopathology and selected cognitive outcomes, whereas others have found no significant benefit over the placebo [115,116]. These approaches therefore remain insufficiently established for routine use and may ultimately require biologically informed patient stratification to identify individuals most likely to benefit. Their greatest value may therefore lie in biologically stratified or adjunctive approaches rather than universal treatment strategies. Collectively, the contrasting trajectories of muscarinic, TAAR1, and glutamatergic drug development underscore a central translational principle: identifying a biologically plausible target is only the first step; successful precision treatment will also require the demonstration of target engagement, identification of responsive patient subgroups, and clinical outcome measures aligned with the biological mechanism being targeted.

### 4.3. Adjunctive Non-Pharmacological Interventions for Cognition and Functional Recovery

Pharmacological symptom control does not necessarily translate into the recovery of cognitive and functional capacity, highlighting the importance of adjunctive non-pharmacological interventions in schizophrenia. Cognitive remediation (CR) has among the strongest evidence bases in this context and uses structured behavioral training to improve cognitive processes and facilitate their transfer to everyday functioning. A large systematic review and meta-analysis of 130 randomized clinical trials involving 8851 participants demonstrated significant, although modest, benefits of CR for global cognition (Cohen’s d = 0.29, 95% CI 0.24–0.34) and overall functioning (d = 0.22, 95% CI 0.16–0.29), with greater effects when CR was delivered by a trained therapist and integrated with psychiatric rehabilitation [117]. These findings suggest that CR is most effective when cognitive training is embedded within broader rehabilitation rather than implemented as an isolated computerized intervention.

Physical exercise represents another accessible adjunctive strategy with potential benefits extending across psychiatric symptoms, cognition, and physical health. A 2024 meta-analysis of 17 studies involving 973 patients found that adjunctive aerobic exercise improved overall psychopathology and negative symptoms, although substantial heterogeneity in intervention protocols remains [118]. More specifically, a systematic review and meta-analysis of 22 studies involving 1066 participants reported improvement in global cognition following physical exercise, with significant effects on processing speed, attention, and visual learning and memory, but not consistently across all cognitive domains [119]. Exercise should therefore be viewed as a multimodal adjunct rather than a symptom-specific treatment, with potential advantages for both neurocognitive and cardiometabolic health.

Non-invasive brain stimulation, particularly repetitive transcranial magnetic stimulation (rTMS), has also been investigated for symptom dimensions that respond incompletely to antipsychotic medication. Evidence is strongest for high-frequency stimulation of the left DLPFC for negative symptoms, although effect sizes and optimal stimulation parameters vary across studies. An umbrella review of systematic reviews and meta-analyses concluded that high-frequency rTMS targeting the DLPFC may improve negative symptoms, particularly when delivered at intensities above 100% of motor threshold for at least three weeks, whereas evidence for cognitive improvement remains less consistent [120]. A network meta-analysis of 48 randomized trials involving 2211 participants similarly identified several excitatory non-invasive brain stimulation approaches, including high-frequency rTMS, as associated with reductions in negative symptoms relative to sham stimulation [121].

Other technology-assisted approaches, including virtual reality-based social and functional training, are also being explored, but their evidence base remains less mature than that of CR, exercise, or rTMS. More broadly, these interventions should not be viewed as alternatives to pharmacotherapy but as complementary components of individualized treatment. Their differing therapeutic targets also reinforce the multidimensional framework developed throughout this review: whereas antipsychotic medications primarily reduce psychotic symptoms, CR directly addresses cognitive processes and their functional transfer, exercise may influence cognitive, metabolic, and symptom-related outcomes, and neuromodulation can target dysfunctional cortical circuits. Future treatment models should therefore focus on rational combinations of pharmacological and non-pharmacological interventions selected according to predominant symptom dimensions, cognitive profiles, functional goals, and, ultimately, biologically informed patient stratification.

## 5. Barriers to Mechanism-Informed Care

Despite substantial advances in the understanding of schizophrenia pathophysiology, these insights have not yet translated into consistently improved outcomes in routine clinical practice. Several major barriers continue to limit progress, including treatment resistance, adverse-effect burden, poor medication adherence, and the persistent lack of clinically actionable biomarkers to support patient stratification and precision treatment.

### 5.1. Treatment Resistance and the Clozapine Gap

Treatment resistance remains one of the most important unmet needs in schizophrenia and illustrates the limits of applying a uniform pharmacological strategy to a biologically heterogeneous disorder. Approximately 20–30% of patients show an inadequate response to conventional antipsychotic treatment, although estimates vary according to diagnostic criteria, treatment adequacy, and study population [101,122]. The Treatment Response and Resistance in Psychosis (TRRIP) Working Group defines treatment-resistant schizophrenia (TRS) on the basis of persistent symptoms despite adequate trials of at least two antipsychotics, together with a systematic assessment of treatment duration, dose, adherence, and symptom severity [101]. Importantly, apparent treatment resistance should be distinguished from pseudo-resistance resulting from inadequate treatment exposure, poor adherence, substance use, or other potentially modifiable factors.

Clozapine remains the treatment of choice for TRS and has demonstrated superior efficacy compared with other antipsychotics in appropriately defined treatment-resistant populations [123]. Nevertheless, its use remains substantially lower and later than recommended in many clinical settings. Delays in initiating clozapine are clinically important because patients frequently undergo repeated trials of non-clozapine antipsychotics despite limited evidence that such switching provides substantial benefit once genuine treatment resistance has been established. Barriers to clozapine use include mandatory hematological monitoring, concerns regarding agranulocytosis and myocarditis, metabolic and cardiovascular adverse effects, logistical burden, clinician experience, and patient acceptance [124]. Thus, the “clozapine gap” reflects not only pharmacological limitations but also implementation barriers within real-world care.

Treatment resistance may also reflect neurobiological heterogeneity rather than simply greater severity of the same dopaminergic abnormality. Molecular imaging studies have suggested that some patients with TRS do not exhibit the elevated presynaptic striatal dopamine synthesis capacity typically associated with treatment-responsive schizophrenia, raising the possibility of a relatively “non-dopaminergic” subgroup [125,126]. Although this distinction should not be interpreted as a definitive biological classification, it provides an important conceptual explanation for why further intensification of D2 receptor blockade may offer limited benefit in some patients. Glutamatergic, immune, synaptic, and other biological abnormalities may contribute differently across individuals, reinforcing the need to move beyond treatment selection based primarily on clinical diagnosis.

Accordingly, improving outcomes in TRS will require both a better implementation of established treatments and more precise biological stratification. Earlier identification of genuine treatment resistance, timely initiation of clozapine, and differentiation of pseudo-resistance from biologically distinct forms of non-response are immediate clinical priorities. In parallel, biomarkers capable of identifying dopamine-responsive and non-dopaminergic disease profiles could eventually guide treatment selection before multiple ineffective medication trials occur. TRS therefore provides a particularly clear example of why precision psychiatry must integrate clinical trajectories with underlying biological mechanisms rather than relying on repeated empirical switching between pharmacologically similar treatments.

### 5.2. Adherence, Adverse Effects, and Long-Term Treatment Challenges

Even when an effective antipsychotic is identified, maintaining long-term treatment effectiveness remains a major challenge in schizophrenia. Continuous antipsychotic treatment is important for relapse prevention, yet treatment discontinuation and suboptimal adherence frequently compromise outcomes in routine clinical practice. Non-adherence is associated with an increased risk of relapse and hospitalization, and is influenced by multiple interacting factors, including limited insight, negative attitudes toward medication, substance use, adverse effects, and the quality of the therapeutic relationship. Accordingly, adherence should not be viewed simply as a patient-level behavioral problem but as a dynamic outcome shaped by treatment tolerability, illness characteristics, and the clinical environment.

The adverse-effect burden of antipsychotic treatment represents an important contributor to long-term treatment acceptability. Antipsychotics differ substantially in their propensity to cause weight gain and metabolic abnormalities, extrapyramidal symptoms, sedation, hyperprolactinemia, and other adverse effects, creating clinically meaningful trade-offs between efficacy and tolerability [98,127]. These effects are particularly relevant because schizophrenia commonly requires prolonged or lifelong treatment, allowing even moderate adverse effects to accumulate into substantial cardiometabolic and functional burden. Emerging evidence also suggests that antipsychotic-associated metabolic effects may interact with the gut microbiome. Antipsychotic exposure has been associated with alterations in microbial composition and metabolic function, which may in turn relate to changes in body weight and metabolic parameters, although the direction and causal significance of these relationships remain uncertain [95]. These findings raise the possibility that host–microbiome interactions contribute to interindividual variability in metabolic treatment burden, but their clinical utility for predicting or managing antipsychotic-related adverse effects has not yet been established. Importantly, adverse-effect profiles vary both across drugs and among individuals receiving the same drug, emphasizing that treatment selection should consider not only symptom reduction but also comorbidities, previous treatment response, patient priorities, and long-term acceptability.

Long-acting injectable antipsychotics (LAIs) provide one strategy for addressing adherence-related treatment failure by reducing the need for daily medication and making treatment interruption more readily identifiable. A large comparative meta-analysis encompassing randomized, cohort, and pre–post studies found that LAIs were associated with a lower risk of hospitalization or relapse than oral antipsychotics across all three study designs, although the magnitude of benefit differed substantially according to study design. A network meta-analysis of 92 randomized trials involving 22,645 participants similarly supported both oral and LAI antipsychotics for relapse prevention, while highlighting differences among individual treatments [128]. LAIs should therefore not be regarded as universally superior to oral therapy, but rather as an important option when adherence, relapse prevention, treatment preference, or continuity of care are major concerns.

Optimizing long-term treatment consequently requires more than selecting the drug with the greatest average efficacy. Shared decision-making, systematic monitoring and management of adverse effects, consideration of LAI formulations when appropriate, and the incorporation of patient preferences are essential for sustaining treatment engagement. At the same time, substantial interindividual variability in both therapeutic response and adverse-effect susceptibility demonstrates the limitations of population-level treatment algorithms. The ability to predict which patients are most likely to respond to a particular treatment—and which are most vulnerable to specific adverse effects—therefore represents an important prerequisite for moving from empirical prescribing toward precision psychiatry.

### 5.3. Biomarker Gaps and the Transition Toward Precision Psychiatry

The marked clinical and biological heterogeneity of schizophrenia remains a major obstacle to translating advances in pathophysiology into individualized treatment. Patients sharing the same diagnosis can differ substantially in symptom dimensions, illness trajectories, treatment response, adverse-effect susceptibility, and underlying biological alterations. Although candidate biomarkers have been identified across the genomic, molecular, neuroimaging, electrophysiological, and other domains, no single biomarker currently provides sufficient reproducibility and clinical utility to guide routine treatment selection in schizophrenia [129,130]. This limitation is consistent with the view that schizophrenia reflects partially overlapping biological processes rather than a uniform pathophysiological entity, shifting the objective from identifying a universal disease marker toward defining clinically meaningful biological subgroups.

Single-modality biomarkers illustrate both the progress and the limitations of this approach. Polygenic risk scores capture part of the inherited liability to schizophrenia, but their current predictive value is insufficient to determine treatment selection at the individual level [131]. Integrating genetic liability with neuroimaging may provide additional biological information, although associations between schizophrenia polygenic risk and structural or functional brain measures remain heterogeneous [132]. Neuroimaging measures of brain structure, connectivity, neurochemistry, and receptor function likewise have considerable potential for characterizing disease mechanisms and pharmacological target engagement, but validated imaging biomarkers for routine clinical decision-making remain lacking [133]. Similarly, inflammatory abnormalities are reproducibly detectable at the group level in subsets of patients, yet substantial heterogeneity limits their translation into individual-level diagnostic or treatment-response markers [134,135]. These findings suggest that the most clinically useful biomarkers may be those linked to specific outcomes—such as treatment response, treatment resistance, relapse, cognitive impairment, or adverse-effect susceptibility—rather than diagnosis alone.

Treatment-response prediction represents a particularly important test of whether candidate biomarkers can become clinically actionable. Functional connectivity measures have been associated with subsequent antipsychotic response, but differences in imaging acquisition, analytical pipelines, patient populations, and outcome definitions have limited reproducibility across studies [136]. Electrophysiological measures provide a complementary and potentially more scalable approach, with pharmaco-EEG studies identifying candidate changes associated with antipsychotic response; however, methodological heterogeneity and limited independent replication currently preclude routine clinical implementation [137]. Pharmacogenomic approaches similarly offer the possibility of matching treatment to individual genetic profiles, but current randomized evidence remains limited and inconsistent, and the certainty of evidence supporting improved clinical outcomes remains low [138]. Thus, a statistically significant association with treatment outcome should not itself be regarded as evidence of clinical utility.

The increasing availability of multimodal datasets has stimulated the use of machine-learning and artificial-intelligence approaches to integrate complementary sources of information and identify predictive patterns that may not be apparent from individual variables. Beyond clinical prediction, these approaches may also contribute to etiological research by integrating genomic, transcriptomic, neuroimaging, environmental, and longitudinal clinical data to identify cross-modal patterns and biologically distinct disease trajectories. Such analyses may help uncover gene–environment interactions and define mechanistically informative patient subgroups that are difficult to resolve using single-domain approaches. However, AI-derived associations should be regarded primarily as tools for hypothesis generation and prioritization rather than evidence of causality, which requires validation through longitudinal, genetic, and experimental studies [139,140]. A systematic review of machine-learning studies predicting pharmacological outcomes in psychosis identified 28 studies, but only five incorporated multiple data modalities, while small sample sizes, methodological heterogeneity, and limited independent validation remained common limitations [141]. More recent meta-analytic evidence suggests that AI-based models can achieve moderate performance in predicting schizophrenia treatment outcomes, but substantial between-study heterogeneity and concerns regarding generalizability remain [142]. Importantly, high predictive accuracy within a development dataset does not establish clinical readiness. Future models will require prospective testing, external validation across independent and demographically diverse populations, transparent reporting, and demonstration that model-guided decisions improve outcomes beyond conventional clinical assessment.

Digital phenotyping may add a longitudinal dimension to precision psychiatry by capturing behavioral and functional changes outside conventional clinical encounters. Smartphone- and wearable-derived measures can characterize domains such as mobility, sleep, activity, cognition, communication patterns, and self-reported experiences, potentially enabling more continuous assessment of symptom dynamics and relapse risk. A recent systematic review encompassing 142 studies and 6294 participants found promising digital signals across schizophrenia-spectrum disorders but also substantial heterogeneity in methods, limited standardization, and important gaps in validation against established clinical measures [143]. Reviews of smartphone-based monitoring in schizophrenia similarly indicate rapid technological development but limited replication across platforms and considerable variability in engagement, outcome definitions, and implementation [144]. Digital measures should therefore be considered complementary to, rather than replacements for, structured clinical evaluation.

Precision psychiatry should ultimately be defined not by the complexity of its technologies but by its ability to improve clinically meaningful decisions. The most useful future models will likely integrate clinical phenotype and treatment history with selected genomic, molecular, neuroimaging, electrophysiological, and longitudinal digital information, rather than relying on any single biomarker. Such models must also remain interpretable, scalable, and sufficiently practical for real-world implementation. Critically, prediction alone is insufficient: candidate biomarkers must demonstrate analytical validity, reproducibility, prospective clinical utility, and incremental value over existing clinical assessment before they can guide treatment. The ultimate goal is therefore not simply to classify schizophrenia into increasingly complex biological subtypes, but to determine which patient is most likely to benefit from which intervention, at what stage of illness, and with an acceptable balance between efficacy and adverse effects.

## 6. Conclusions and Future Directions

Schizophrenia should no longer be conceptualized solely as a disorder of dopaminergic dysfunction but rather as a complex neuropsychiatric disorder arising from the convergence of multiple interacting biological systems. Genetic susceptibility, environmental exposures, neurotransmitter imbalance, neuroimmune activation, kynurenine pathway dysregulation, glial dysfunction, cellular stress and mitochondrial dysfunction, large-scale brain network abnormalities, and gut–brain communication collectively contribute to the heterogeneous clinical manifestations of schizophrenia. Viewing these mechanisms within an integrated systems-level framework provides a more comprehensive explanation for persistent negative symptoms, cognitive impairment, treatment resistance, and the marked variability in therapeutic response than any single pathogenic hypothesis alone.

This converging neurobiological perspective also has important implications for therapeutic development. While D2 receptor-based antipsychotics remain central to the control of positive symptoms, they address only part of the biological complexity underlying schizophrenia. Recent advances—including non-dopaminergic pharmacological therapies, neuromodulation, cognitive rehabilitation, and other recovery-oriented interventions—reflect a broader transition toward mechanism-informed treatment strategies. Nevertheless, substantial barriers continue to impede clinical translation, including treatment resistance, medication non-adherence, long-term tolerability, and the absence of validated, clinically actionable biomarkers capable of supporting biologically informed patient stratification.

Looking forward, future research should move beyond investigating isolated molecular pathways and instead prioritize the integration of multimodal biological information across the genetic, transcriptomic, immune, metabolic, neuroimaging, and digital phenotyping domains. Combining these complementary data with advances in artificial intelligence and computational modeling may facilitate the identification of biologically distinct patient subgroups and improve the prediction of treatment response and disease trajectory. Such integrative approaches will be essential for transforming precision psychiatry from a conceptual framework into a practical clinical reality.

Ultimately, the greatest challenge for schizophrenia research is no longer simply identifying additional pathogenic mechanisms, but understanding how multiple interconnected biological systems interact to shape disease heterogeneity across individual patients. Bridging this gap between biological discovery and clinical implementation will be critical for developing more personalized, mechanism-informed, and clinically meaningful interventions. As our understanding of converging neurobiological mechanisms continues to evolve, it may provide a foundation for more effective precision psychiatry and improved long-term outcomes for individuals living with schizophrenia.

## Data Availability

No new data were created or analyzed in this study. Data sharing is not applicable to this article.
